# Marginal Zone B Cells Induce Alloantibody Formation Following RBC Transfusion

**DOI:** 10.3389/fimmu.2018.02516

**Published:** 2018-11-16

**Authors:** Seema R. Patel, David R. Gibb, Kathryn Girard-Pierce, Xiaoxi Zhou, Lilian Cataldi Rodrigues, Connie M. Arthur, Ashley L. Bennett, Ryan P. Jajosky, Megan Fuller, Cheryl L. Maier, Patricia E. Zerra, Satheesh Chonat, Nicole H. Smith, Christopher A. Tormey, Jeanne E. Hendrickson, Sean R. Stowell

**Affiliations:** ^1^Department of Laboratory Medicine and Pathology, Center for Transfusion Medicine and Cellular Therapies, Emory University School of Medicine, Atlanta, GA, United States; ^2^Department of Laboratory Medicine, Yale University School of Medicine, New Haven, CT, United States; ^3^Aflac Cancer and Blood Disorders Center, Children's Healthcare of Atlanta/Emory University School of Medicine, Atlanta, GA, United States

**Keywords:** marginal zone (MZ) B cells, RBC alloimmunization, follicular B cells, CD4 T cells, splenic marginal zone

## Abstract

Red blood cell (RBC) alloimmunization represents a significant immunological challenge for some patients. While a variety of immune constituents likely contribute to the initiation and orchestration of alloantibodies to RBC antigens, identification of key immune factors that initiate alloantibody formation may aid in the development of a therapeutic modality to minimize or prevent this process. To define the immune factors that may be important in driving alloimmunization to an RBC antigen, we determined the specific immune compartment and distinct cells that may initially engage transfused RBCs and facilitate subsequent alloimmunization. Our findings demonstrate that the splenic compartment is essential for formation of anti-KEL antibodies following KEL RBC transfusion. Within the spleen, transfused KEL RBCs are found within the marginal sinus, where they appear to specifically co-localize with marginal zone (MZ) B cells. Consistent with this, removal of MZ B cells completely prevented alloantibody formation following KEL RBC transfusion. While MZ B cells can mediate a variety of key downstream immune pathways, depletion of follicular B cells or CD4 T cells failed to similarly impact the anti-KEL antibody response, suggesting that MZ B cells may play a key role in the development of anti-KEL IgM and IgG following KEL RBC transfusion. These findings highlight a key contributor to KEL RBC-induced antibody formation, wherein MZ B cells facilitate antibody formation following RBC transfusion.

## Introduction

While red blood cell (RBC) transfusion support is a vital therapy for patients with congenital hemoglobinopathies, transfusion is not without risk. RBCs express a large number of allogeneically distinct antigens that can lead to the development of alloantibodies following transfusion (1–3). Formation of alloantibodies can directly increase morbidity and mortality of transfusion dependent patients by increasing the probability of hemolytic transfusion reactions (3–6), compromising the therapeutic efficacy of transfused cells and decreasing the availability of compatible RBCs for future transfusions (2, 7–10). While antigen-matching protocols can reduce alloimmunization risk, recent studies demonstrate that these approaches unfortunately fail to successfully eliminate alloantibody formation (11). Furthermore, although prophylactic use of anti-D globulin can prevent anti-D antibody formation following exposure to RhD positive RBCs, there are currently no therapeutic modalities that actively prevent or minimize the rate of RBC alloimmunization following therapeutic transfusion (3, 12, 13). The inability to prevent RBC alloimmunization largely stems from a fundamental lack of understanding regarding key pathways that regulate this process. Understanding the mechanism(s) by which alloantibodies to RBC antigens develop may aid in the identification of key targets that can be used to inhibit RBC alloimmunization in chronically transfused individuals.

Although several immune constituents appear to play a key role in the development of a humoral immune response to blood borne antigens (14), the exact role each of these populations play in the induction of a humoral immune response to RBC alloantigens remains incompletely defined. As HLA Class II alleles have been found to correlate with RBC alloimmunization (15–24) and CD4 T cells from alloimmunized individuals have been identified within some RBC antigens (15, 25), key aspects of T cell responses to different RBC alloantigens has been the primary focus of mechanistic studies on RBC alloimmunization. However, while CD4 T cells may be important for the formation of a robust antibody response to some RBC alloantigens, the ability to develop an immune response to a given immunogen is thought to first require the initial capture and subsequent response to an antigenic determinant by other immune populations. Indeed, a number of innate immune constituents within the marginal sinus of the spleen possess the ability to trap circulating antigen for immune recognition and antigenic removal, as well as transport of antigenic cargo to the B cell follicle (26–28). In particular, marginal zone (MZ) B cells are a specialized innate-like B cell population that can be a key contributor to humoral immunity to blood borne antigens (29, 30). MZ B cells are the only cells known within the marginal sinus to not only possess antigen specific receptors (31, 32), but also can mediate prompt detection of blood borne antigens and immediate antibody production. Several studies demonstrate that MZ B cells can also contribute to canonical follicular B cell responses by delivering antigen to the follicle (28, 29, 33, 34) and/or activating CD4 T helper cells (35).

Given the importance of identifying key initiating factors in regulating alloantibody formation, we sought to characterize the immune cells responsible for the initial recognition and response to RBC alloantigens following transfusion. As mechanistic studies in humans defining early localization and immune responses to RBC alloantigens are not justifiable and mice do not inherently express RBC polymorphisms capable of inducing an alloantibody response following RBC exposure, a mouse model of RBC alloimmunization was recently generated by expressing the human KEL antigen, one of the most common RBC alloantigens implicated in hemolytic transfusion reactions (36–41), on murine RBCs (42–46). Using this model system, we found that not only is the spleen required for formation of alloantibodies to KEL but also that KEL RBCs co-localize with splenic MZ B cells after transfusion, suggesting a potential role for this distinct B cell population in alloantibody formation. Consistent with this, removal of MZ B cells specifically inhibited alloimmunization following KEL RBC transfusion. While MZ B cells possess the ability to coordinate a variety of down-stream immune pathways, removal of follicular B cells and CD4 T cells failed to significantly impact RBC alloantibody formation. These results suggest that MZ B cells may not only play a role in initiating alloantibody formation following KEL RBC exposure, but that in addition to follicular B cells, MZ B cells may be intrinsically capable of generating an IgG response against the KEL antigen.

## Materials and methods

### Mice

Female B6 (C57BL/6; H-2^b^) recipients were purchased from the National Cancer Institute (Frederick, MD) or Charles River (Wilmington, MA). Female CD45.1^+^ B6 (C57BL/6-Ly5.1; H-2^b^) recipients were also purchased from Charles River. CD19Cre (B6.129P2(C)-*Cd19*^*tm*1(*cre*)*Cgn*^*/J*; H-2^b^), Notch2flx (B6.129S-*Notch2*^*tm*3*Grid*^*/J*; H-2^b^), and IFNAR knockout (B6.129S2-*Ifnar1*^*tm*1*Agt*^*/*Mmjax; IFNAR1 KO; H-2^b^) mice were purchased from Jackson Laboratories (Bar Harbor, ME). CD19Cre mice were bred to Notch2flx recipients to generate (CD19Cre/^+^ x Notch2^flx/+^) recipients that were then bred back to Notch2flx mice to generate (Notch2^flx/flx^ x CD19Cre^/+^) MZ B cell KO recipients. Transgenic KEL (H-2^b^) donors (42) were a generous gift from Dr. James C. Zimring [BloodWorks Northwest, Seattle, WA]. All mice were used at 8–12 weeks of age. Mice were housed and bred in Emory University Department of Animal Resources facilities, and all procedures were performed according to approved Institutional Care and Use Committee (IACUC) protocols.

### Antibodies for flow cytometry

APC anti-mouse CD4 (clone: RM4-5), BV605 anti-mouse CD4 (clone: GK1.5), FITC anti-mouse CD3ε, PE anti-mouse CD8α, V500 rat anti-mouse B220, FITC anti-mouse CD45R/B220, APC anti-mouse IgD, APC anti-mouse CD21/CD35, PE anti-mouse CD23, FITC anti-mouse CD8, BV786 anti-mouse CD3, PE CF594 anti-mouse CD23, APC Cy7 anti-mouse CD21, FITC anti-mouse Gr1, APC anti-mouse CD11b, PE anti-mouse F4/80, FITC anti-mouse I-A/E, and APC streptavidin were bought from BD bioscience (San Jose, CA). Biotinylated anti-mouse C3 was obtained from Cedarlane (Burlington, Canada), and APC anti-mouse IgG, APC anti-mouse IgM and FITC anti-mouse IgM were purchased from Jackson Immunoresearch (West Grove, PA). PE anti-mouse CD1d and Zombie yellow live dead was purchased from Biolegend (San diego, CA).

### Cellular depletion and RBC transfusion

MZ B cell depletion was achieved by treating B6 recipients 4 and 2 days prior to transfusion with intra-peritoneal injections of 100 μg monoclonal anti-mouse CD11a antibody (clone: M17/4; Bioxcell, West Lebanon, NH) combined with 100 μg monoclonal anti-mouse CD49d antibody (clone: PS/2; Bioxcell) diluted in PBS (47–49). CD4 T cell depletion was achieved by two intra-peritoneal injections of 250 μg monoclonal anti-mouse CD4 depleting antibody (clone: GK1.5; Bioxcell) diluted in PBS, 4 and 2 days prior to transfusion (50). Follicular B cell depletion was attained through a single tail vein injection of 250 μg monoclonal anti-mouse CD20 IgG1 depleting antibody (clone: 18B12; Biogen Idec, Cambridge, MA) 14 days prior to transfusion (51). To control for non-specific effects of antibody treatment, separate B6 recipients were administered isotype control antibodies: Rat IgG2b mixed with Rat IgG2a (clones: LTF2 and 2A3; MZ B cell depletion control; Bioxcell), Rat IgG2b (clone: LTF2; CD4 T cell depletion control; Bioxcell), or mouse IgG1 (clone: MOPC-21; follicular B cell depletion control; Biogen Idec). Depletion of MZ B cells and follicular B cells was assessed by staining splenocytes with FITC rat anti-mouse CD45R/B220 + PE rat anti-mouse CD23 + APC rat anti-mouse CD21. MZ B cells and follicular B cells were quantified in the spleen by gating on B220^+^ CD23^−^ CD21^hi^ or B220^+^ CD23^hi^ CD21^lo/−^, respectively. To examine the impact of MZ B cell depletion on follicular B cells and T cells, samples were also stained with BV786 anti-mouse CD3 + PE anti-mouse CD8 + APC anti-mouse CD4 + V500 anti-mouse CD45R/B220 + PE CF594 anti-mouse CD23 + APC Cy7 anti-mouse CD21. To determine if MZ B cell depletion impacted macrophages, dendritic cells and neutrophils, samples were also stained with APC anti-mouse CD11b + PE anti-mouse F4/80 (for macrophages), APC anti-mouse CD11c + FITC anti-mouse I-A/E^b^ (for dendritic cells) or APC anti-mouse CD11b + FITC anti-mouse Gr1 (for neutrophils). Efficacy of CD4 T cell depletion was assessed by peripheral blood (prior to transfusion) with FITC rat anti-mouse CD3ε + PE rat anti-mouse CD8α + APC rat anti-mouse CD4 (clone: RM-45) ± BV605 rat anti-mouse CD4 (clone: GK1.5). Percent CD4 T cells were computed by gating on CD3^+^ CD4^+^ CD8^−^ T cells. Samples were run on a 4 color BD FACSCalibur or BD LSRII and analyzed using FlowJo. Donor KEL whole blood was collected 1:8 into acid citrate dextrose (ACD, Vacutainer, Franklin Lakes, NJ) and washed 3 times with PBS as outlined previously. Recipients were then transfused via the lateral tail vein with 50 μl of packed KEL RBCs diluted in PBS to 300 μl total volume (equivalent to 1 human unit) as done previously (43–45, 52–57).

### Survival of RBCs, direct antiglobulin test and complement component 3 (C3) fixation on transfused RBCs *in vivo*

Wild type B6 or transgenic KEL whole blood was collected 1:8 into ACD and washed 3 times with PBS. Resulting B6 packed RBCs were labeled with 3, 39-dihexadecyloxacarbocyanine perchlorate (DiO; Molecular Probes, Eugene, OR), while KEL packed RBCs were labeled with chloromethylbenzamido 1, 19-dioctadecyl-3, 3, 39, 39-tetramethylindocarbocyanine perchlorate (CM-DiI; Molecular Probes), as previously described (43–45, 52–57). Briefly, 1 mL packed RBCs were diluted 1:10 in PBS. DiO or DiI was next added to the respective RBC samples at a 1:100 dilution. Samples were incubated for 30 min at 37°C and then washed 3 times to remove any unbound dye. Both populations were subsequently mixed at a 1:1 ratio and recipients were transfused via lateral tail vein with 50 μl of each type of blood diluted in PBS to a total volume of 300 μl. At 10 min, and on days 3, 5, 7, and 14 post transfusion, KEL RBC survival was examined. Survival of transfused KEL-DiI RBCs was measured by normalizing percent KEL-DiI RBCs to tracer B6-DiO RBCs. C3 fixation on transfused KEL-DiI RBCs was assessed on days 5 and 14 post transfusion by incubating samples for 30 min at 4°C with biotinylated anti-mouse C3 antibody diluted 1:100 in PBS + 2% bovine serum albumin (BSA) buffer, followed by APC streptavidin diluted 1:100 in PBS + 2% BSA buffer for 30 min at 4°C. Antibody deposition on transfused KEL-DiI RBCs was examined on days 5 and 14 post transfusion by a direct antiglobulin test wherein samples were stained for 30 min at 4°C with APC anti-mouse IgG or APC anti-mouse IgM diluted 1:100 in PBS + 2% BSA buffer. All samples were run on a 4-color BD FACSCalibur and analyzed by FlowJo; mean fluorescence intensity (MFI) was used to measure antibody binding and C3 fixation on transfused KEL-DiI RBCs.

### Splenectomy

B6 recipients were splenectomized as previously described (58). Briefly, recipients were anesthetized and shaved prior to surgery. Using sterile surgical techniques, the spleen was gently extracted and blood vessels were cauterized with a bovie. Control mice were either un-manipulated or underwent sham operations. Two weeks post-surgery, sutures were removed and recipients were transfused with 50 μl packed KEL RBCs diluted in PBS to 300 μl total volume.

### Confocal microscopy

Recipients were transfused via lateral vein with PBS or 50 μl of packed KEL-DiO RBCs diluted to 300 μl total volume in PBS. 1, 3, 5, and 7-days post transfusion, spleens were quick frozen in isopentane on dry ice using TissueTek optical cutting temperature freezing medium (VWR Scientific, Randor, PA). 7 μm thick frozen sections were cut and fixed in acetone for 20 min at −20°C. Fixed sections were then washed 3 times in PBS and incubated in 0.5% blocking buffer [PBS + 0.5% fetal bovine serum (FBS)] for 2 h at room temperature. The blocking buffer was then removed and sections were stained with PE anti-mouse CD1d + Alexa Fluor 647 anti-mouse IgD diluted in blocking buffer for 1 h at room temperature. Sections were then washed 3 times with PBS and mounted using Prolong Gold anti-fade mountant (ThermoFisher Scientific, Waltham, MA). Images were acquired using the HC Plan Fluotar 10X (0.3 NA air, WD 11.0 mm) or HC Plan APO CS2 40X (1.3 NA oil, WD 0.24 mm) objective on a Leica SP8 multiphoton confocal microscope. Images were then analyzed using the Leica application suite (LAS) Advanced Fluorescence lite software.

### Bone marrow chimera

Chimeric recipients were generated as previously described (59). CD45.1^+^ B6 recipients were irradiated with two doses of 6.35 Gy 3 h apart using an X-RAD 320, Precision x–ray irradiator. 2 to 4 h post irradiation, bone marrow was harvested from CD45.2^+^ B6, CD45.2^+^ CD19Cre^+/−^, CD45.2^+^ (Notch2^flx/flx^ × CD19Cre^/+^), and CD45.2^+^ IFNAR1 KO donors as previously described. Briefly, marrow was flushed from the femurs of donors using 5% FBS in RPMI and a 25G needle. The cells were then passed through an 18G needle to homogenize the marrow. The cells were next filtered through a 70 μm filter to remove debris and washed two times with 1x PBS. Recipients were then transfused into indicated recipients via the lateral tail vein (3 × 10^6^ bone marrow cells total). Reconstituted recipients were transfused with KEL RBCs by lateral tail vein injection 8 to 9 weeks post transplantation.

### Seroanalysis

Serum collected 5, 7, and 14 days post transfusion was evaluated for anti-KEL antibodies by indirect immunofluorescence staining, as previously described (43–45, 55–57, 60–63). Briefly, neat serum was incubated with packed KEL or B6 RBCs for 15 min at room temperature. Samples were then washed 3 times with FACS buffer (PBS + 2% BSA + 0.9 g EDTA), and incubated for 30 min in APC anti-mouse IgG + FITC anti-mouse IgM diluted 1:100 in FACS buffer. Samples were run on a 4-color BD FACSCalibur and analyzed using FlowJo; MFI of indicated fluorophores was used to measure the amount of antigen specific antibody subsets present in the serum. While the antibodies detected following KEL RBC transfusion are not technically “alloantibodies,” they have been commonly referred to as alloantibodies in previous work, as the KEL system is a model of RBC alloimmunization (44–46, 59). In an effort to continue to provide uniformity of nomenclature within the field, we will continue to use this term to refer to antibodies generated in response to KEL RBC transfusion in the present work.

### Factor VIII (FVIII) immunization and Anti-FVIII antibody detection

B6 recipients were depleted of MZ B cells 4 and 2 days prior to administration of 2 μg FVIII, as well as 10 and 20 days post the initial FVIII injection, as previously described (49). FVIII was given once a week for 4 weeks via retro-orbital injection as outlined previously. Plasma was harvested 7 days post the last immunization. An enzyme-linked immunosorbent assay (ELISA) was performed to detect anti-FVIII IgG, as previously described (49, 64–66). Briefly, ELISA plates were coated with FVIII diluted in coating buffer (PBS + 0.05% sodium azide) overnight at 4°C. Plates were then blocked for 2 h at room temperature. Following the 2-h incubation, plates were washed and incubated for 1 h at room temperature with plasma serially diluted in blocking buffer. Following an hour, samples were washed and incubated for 1 h at room temperature with secondary anti-mouse IgG HRP diluted 1:1000 in blocking buffer. Samples were then washed, substrate was added and samples were read at 405 nm on an ELISA plate reader (Spectramax plus).

### Statistics

Statistical analysis was performed using one-way ANOVA with Tukey's post-test or two-way ANOVA with Tukey's post-test. Significance was determined by a *P-*value less than 0.05.

## Results

### An intact spleen is required for KEL alloimmunization

**Figure 1:**
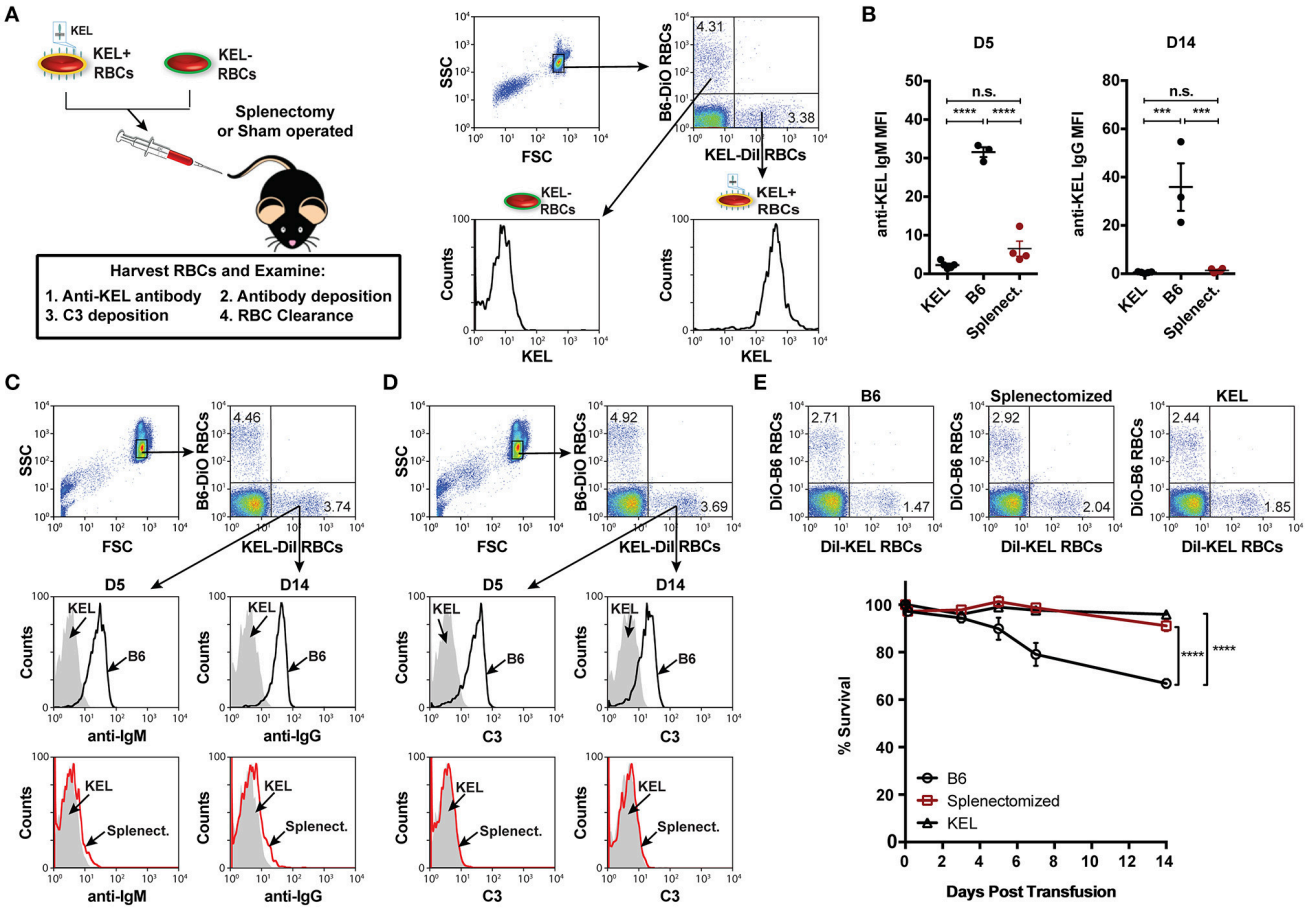
Alloimmunization to KEL is dependent on an intact spleen. **(A)** Experimental schematic. B6 recipients negative for KEL were splenectomized (Splenect.) or sham operated (B6) 2 weeks prior to transfusion with a 1:1 mixture of KEL and B6 RBCs labeled with DiI or DiO, respectively. Recipients were then evaluated for anti-KEL antibody production, antibody binding and C3 deposition on transfused KEL-DiI RBCs, as well as survival of transfused KEL-DiI RBCs. **(B)** Anti-KEL IgM and IgG antibody formation in splenectomized (Splenect.), sham operated (B6), and KEL positive recipients 5 (D5) and 14 (D14) days post transfusion, respectively. **(C)** Anti-IgM and IgG deposition on transfused KEL RBCs in splenectomized (Splenect.; red line), sham operated (B6; black line), and KEL positive (gray shade) recipients 5 (D5) and 14 (D14) days post transfusion, respectively. **(D)** C3 fixation on transfused KEL-DiI RBCs in splenectomized (Splenect.; red line), sham operated (B6; black line), and KEL positive (gray shade) recipients 5 (D5) and 14 (D14) days post transfusion. **(E)** Dot plots (day 14) and quantification of KEL-DiI RBC survival in splenectomized, sham operated (B6), and KEL positive recipients at 10 min, and days 3, 5, 7, and 14 post transfusion. Survival of transfused KEL-DiI RBCs was measured by normalizing percent KEL-DiI RBCs to tracer B6-DiO RBCs. Error bars represent mean ± SEM. The mean of each group is depicted as a horizontal line in panel **(B)**. Statistics were generated using a one-way ANOVA with Tukey's post-test in panel **(B)** and in panel **(E)** the depicted statistical value illustrates the statistical analysis for day 14 analyzed by two-way ANOVA with Tukey's post-test. All panels show representative data from experiments reproduced 3 times, with 3-4 mice per group per experiment. ^****^p < 0.0001; ^***^p < 0.001; and n.s., not statistically significant.

To define key initiators in alloantibody formation to the KEL antigen following KEL RBC transfusion, we first sought to determine whether a specific immune compartment was required for the development of anti-KEL alloantibodies. Given the potential role of the spleen in antibody formation against RBC alloantigens (67, 68), we sought to determine whether the spleen was responsible for anti-KEL antibody formation following transfusion of KEL RBCs. To accomplish this, KEL negative B6 recipients were splenectomized or sham operated 2 weeks prior to transfusion of a mixture of KEL RBCs labeled with a lipophilic dye, DiI, and B6 RBCs that were labeled with a fluorescently distinct dye, DiO, to facilitate direct examination of specific changes to KEL RBC survival, antibody binding and complement deposition post-transfusion (Figure 1A). Splenic removal impeded the generation of anti-KEL IgM and IgG alloantibodies (Figure 1B). The reduced level of detectable anti-KEL alloantibodies in splenectomized B6 recipients was not due to sequestration of anti-KEL antibodies on the surface of transfused KEL RBCs, as anti-IgM and anti-IgG alloantibodies were not detectable on KEL RBCs transfused into splenectomized B6 recipients (Figure 1C). Moreover, the decreased level of detectable anti-KEL alloantibodies in splenectomized B6 recipients correlated with a lack of C3 on transfused KEL-DiI RBCs (Figure 1D), as well as no detectable KEL RBC clearance above that of KEL RBCs transfused into KEL positive recipients (Figure 1E). These findings demonstrate that splenic immune constituents are important for the formation of alloantibodies against KEL on transfused RBCs, consistent with previous studies in patients (67, 68).

### Transfused KEL RBCs co-localize with MZ B cells

**Figure 2:**
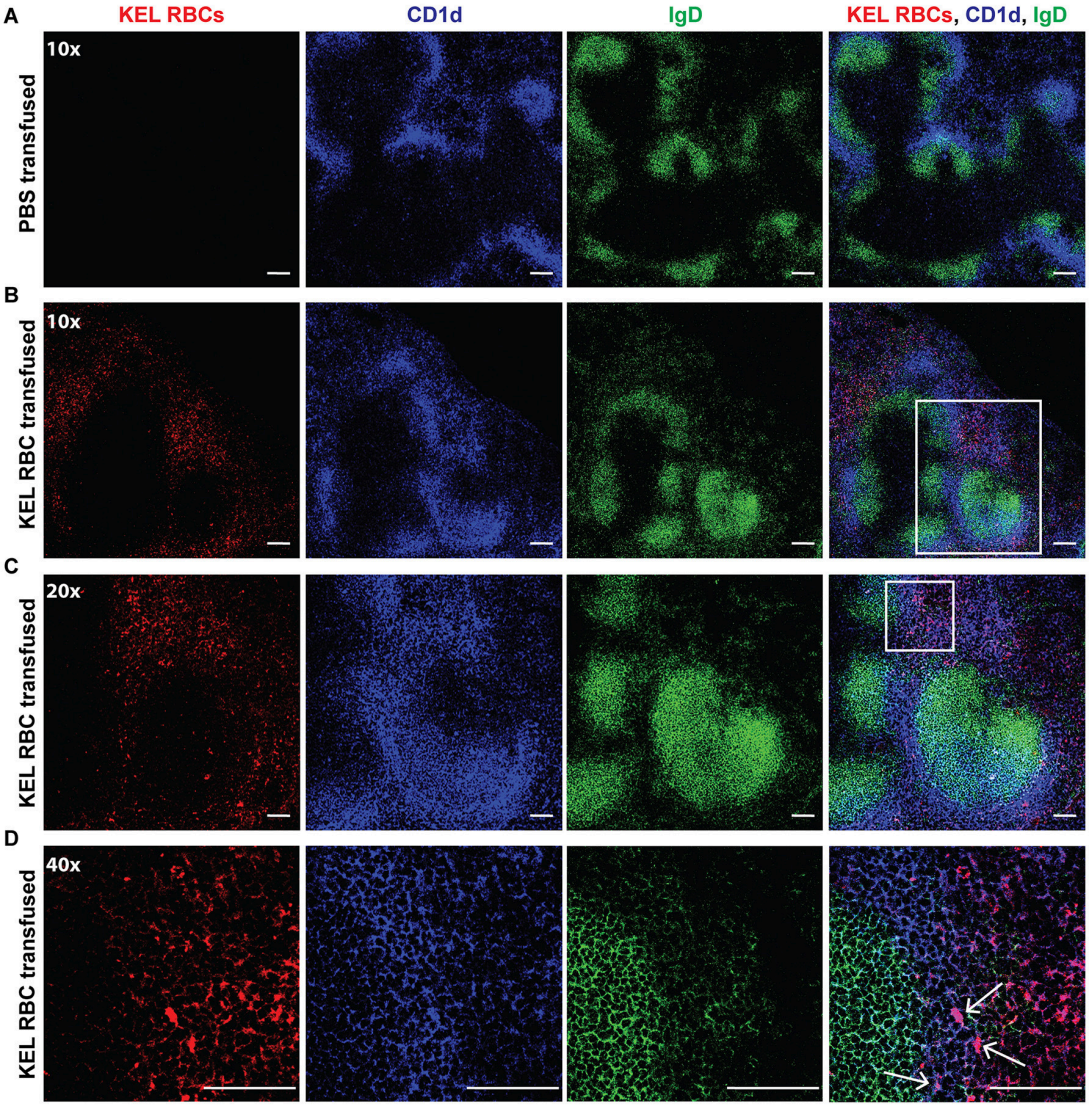
MZ B cells co-localize with transfused KEL RBCs. B6 recipients negative for KEL were transfused with PBS **(A)** or KEL-DiO RBCs **(B**; red**)**, followed by confocal analysis of KEL RBC co-localization with splenic MZ B cells 24 h post transfusion. MZ B cells are identified as IgD (green) dim and CD1d (blue) bright cells, while follicular B cells are distinguished as IgD (green) bright and CD1d dim. White arrows indicate examples of co-localization of MZ B cells and transfused KEL-DiO RBCs. Samples were analyzed using a 10x **(A,B)**, 20x **(C)** or 40x **(D)** objective. Scale bar = 100 μm. All panels show representative data from experiments reproduced 2 times, with 3 mice per group per experiment.

As the spleen contains distinct immune populations capable of facilitating the initial recognition and response to foreign antigen, we first examined transfused KEL RBC localization within the spleen. B6 recipients negative for KEL were transfused with PBS or KEL RBCs labeled with the fluorescent lipophilic dye, DiO. Twenty-Four hours post-transfusion, the spleen was analyzed by confocal microscopy and stained for MZ B cells indicated by CD1d, a non-classical MHC Class I molecule that is expressed by MZ B cells at a greater level than follicular B cells and routinely utilized to specifically detect MZ B cells by confocal microscopy (69–71). As follicular B cells express a greater level of IgD than MZ B cells, IgD was utilized in combination with CD1d to distinguish MZ B cells (CD1d bright, IgD dim) from follicular B cells (CD1d dim/-, IgD bright). KEL-DiO RBCs were found to co-localize with some MZ B cells within 24 h post transfusion (Figure 2). Similarly, KEL-DiO RBCs were found to co-localize with some MZ B cells 3, 5, and 7-days post transfusion, though the amount of co-localization was lower, possibly due to fewer circulating KEL RBCs overtime (Supplementary Figure 1). Together, these data suggest that MZ B cells may be involved in the development of an anti-KEL immune response.

### KEL alloimmunization is MZ B cell dependent

**Figure 3:**
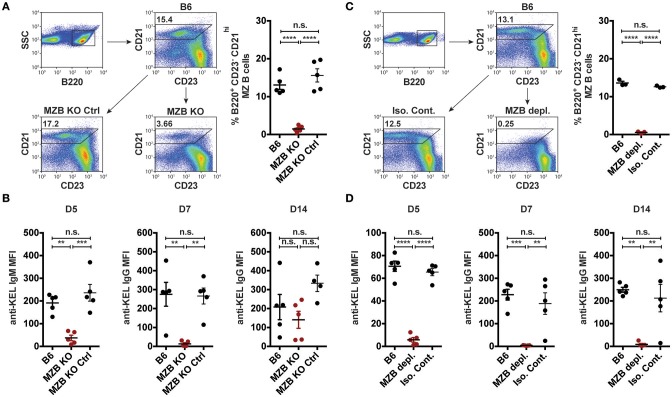
KEL specific alloantibody formation is dependent on MZ B cells. **(A)** Gating strategy and quantification of percent splenic B220^+^ CD21^hi^ CD23^−^ MZ B cells in wild type B6, MZ B cell KO (MZB KO), and MZ B cell KO littermate controls (MZB KO Ctrl). **(B)** Anti-KEL alloantibody formation in B6, MZ B cell KO (MZB KO), and MZ B cell KO littermate control (MZB KO Ctrl) recipients transfused with KEL RBCs. **(C)** Flow plots and quantification of percent splenic B220^+^ CD21^hi^ CD23^−^ MZ B cells in B6 recipients treated with PBS (B6), monoclonal anti-mouse CD11a + anti-mouse CD49d antibody (MZB depl.), or Rat IgG2a + Rat IgG2b isotype control antibody (Iso. Cont.). **(D)** Anti-KEL specific alloantibody formation in PBS (B6), monoclonal anti-mouse CD11a + anti-mouse CD49d antibody (MZB depl.), or Rat IgG2a + Rat IgG2b isotype control antibody (Iso. Cont.) treated recipients transfused with KEL RBCs. Error bars represent mean ± SEM. The mean of each group is depicted as a horizontal line. Statistics were generated using one-way ANOVA with Tukey's post-test. All panels show representative data from experiments reproduced 3 times, with 5 **(A,B,D)** or 3 **(C)** mice per group per experiment. ^****^p < 0.0001; ^***^p < 0.001; ^**^p < 0.01 and n.s, not statistically significant.

Given that transfused KEL RBCs co-localized with MZ B cells following transfusion (Figure 2), we next sought to determine whether MZ B cells coordinate the formation of an alloantibody response to KEL. As targeted deletion of Notch2 in B cells has been shown to specifically reduce MZ B cell numbers (28, 72), we examined the outcome of KEL RBC transfusion in (Notch2^flx/flx^ x CD19Cre^/+^) recipients that possess a reduced number of CD21^hi^ CD23^−^ B220^+^ MZ B cells compared to controls (Figure 3A). Using these conditional knockout mice (MZ B cell KO), we tested the role of MZ B cells in KEL alloimmunization. Serum was collected 5, 7, and 14 days post transfusion of KEL RBCs, and evaluated for anti-KEL alloantibodies. Transfusion of KEL RBCs into recipients with reduced numbers of MZ B cells resulted in a decreased level of anti-KEL IgM compared to wild type B6 and MZ B cell KO littermate controls (Figure 3B). MZ B cell KO recipients generated a delayed anti-KEL IgG response, with IgG alloantibodies reactive to KEL detectable by day 14-post transfusion (Figure 3B). While these findings suggest that MZ B cells may not be essential for alloantibody formation to KEL on transfused RBCs, the delayed anti-KEL alloantibody response observed in MZ B cell KO recipients may also be due to an incomplete deletion of MZ B cells; approximately 20–30% B220^+^ CD21^hi^ CD23^−^ MZ B cells are detectable in the MZ B cell KO (Notch2^flx/flx^ × CD19Cre^/+^) recipients (Figure 3A).

To circumvent this, we next utilized an alternative and well-characterized approach of MZ B cell depletion (47–49). B6 recipients negative for KEL were administered PBS (B6), a mixture of anti-CD11a (clone: M17/4) and anti-mouse CD49d monoclonal antibodies (clone: PS/2), or a combination of Rat IgG2b (clone: LTF2) and Rat IgG2a (clone: 2A3) isotype control antibodies two times, a day apart. Additional treated recipients were included to determine MZ B cell depletion efficacy, which demonstrated that this approach effectively depleted MZ B cells and illustrated no negative impact on the percent frequency of follicular B cells, T cell subsets, macrophages, dendritic cells and neutrophils (Figure 3C, Supplementary Figure 2) (47, 48). Immediately after establishing depletion, recipients were transfused with KEL RBCs and serum was evaluated for anti-KEL IgM and IgG.

**Figure 4:**
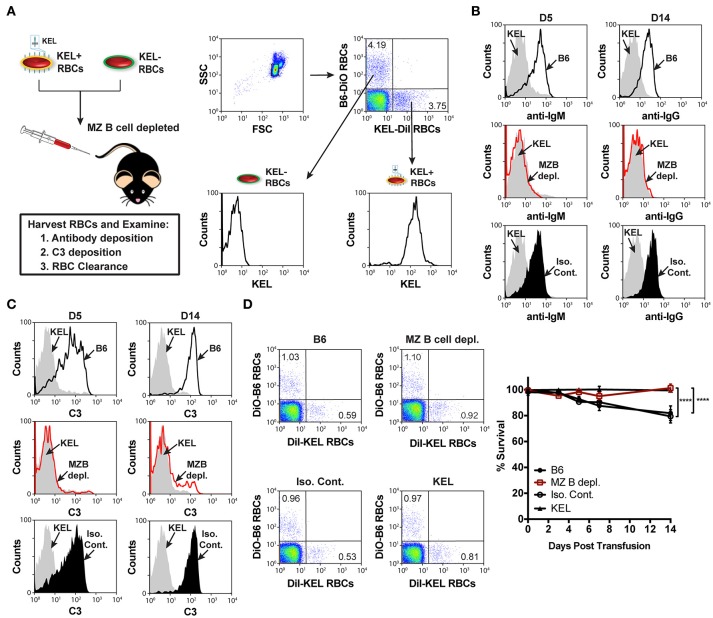
KEL RBCs lack detectable alloantibody and C3 deposition, as well as clearance post transfusion into MZ B cell depleted recipients. **(A)** Experimental schematic and flow cytometric gating strategy used to specifically examine transfused KEL-DiI RBCs. KEL recipients, MZ B cell depleted recipients, and B6 recipients treated with PBS (B6) or isotype control antibodies were transfused with a 1:1 mixture of KEL and B6 RBCs labeled with DiI or DiO, respectively. Recipients were then evaluated for alloantibody and C3 deposition on transfused KEL-DiI RBCs, as well as survival of transfused KEL-DiI RBCs. **(B)** Anti-IgM and anti-IgG antibody binding on transfused KEL-DiI RBCs in PBS treated (B6; black line), MZ B cell depleted (MZ B depl.; red line), isotype control treated (Iso. Cont.; black shade), and KEL positive (gray shade) recipients 5 (D5) and 14 (D14) days post transfusion, respectively. **(C)** C3 fixation on transfused KEL-DiI RBCs in PBS treated (B6; black line), MZ B cell depleted (MZ B depl.; red line), isotype control treated (Iso. Cont.; black shade), and KEL positive (gray shade) recipients 5 (D5) and 14 (D14) days post transfusion. **(D)** Dot plots (day 14) and quantification of KEL-DiI RBC survival in PBS treated (B6), MZ B cell depleted (MZ B depl.), isotype control treated (Iso. Cont.), and KEL positive recipients at 10 min, and days 3, 5, 7, and 14 post transfusion. Survival of transfused KEL-DiI RBCs was measured by normalizing percent KEL-DiI RBCs to tracer B6-DiO RBCs. Error bars represent mean ± SEM in panel **(D)**. The depicted statistical value in panel **(D)** illustrates the statistical analysis for day 14 analyzed by two-way ANOVA with Tukey's post-test. All panels show representative data from experiments reproduced 3 times, with 5 mice per group per experiment. ^****^p < 0.0001.

Similar to the reduced IgM response observed in MZ B cell KO recipients (Figure 3B), MZ B cell depletion significantly abrogated the formation of anti-KEL IgM (Figure 3D). Likewise, the generation of anti-KEL IgG was inhibited in MZ B cell depleted recipients 7 and 14-days post transfusion (Figure 3D). The lack of detectable antibodies in MZ B cell depleted recipients was not due to non-specific effects of the antibody treatment, as isotype control treated recipients generated KEL reactive antibodies comparable to PBS treated B6 recipients (Figure 3D). The requirement of MZ B cells in formation of alloantibodies to the KEL antigen on transfused RBCs was not unique to intravenous exposure to an RBC alloantigen, as MZ B cells were also found to be required for production of alloantibodies to a non-RBC blood borne antigen, factor VIII (Supplementary Figure 3). To determine whether the reduced level of detectable anti-KEL alloantibodies in MZ B cell depleted B6 recipients was due to sequestration of anti-KEL antibodies on transfused KEL RBCs, B6 recipients depleted of MZ B cells were transfused with KEL RBCs labeled with a lipophilic dye, DiI, and B6 RBCs labeled with a fluorescently distinct dye, DiO, to facilitate direct examination of specific changes to antibody and complement deposition as well as KEL RBC survival post transfusion (Figure 4A). Consistent with the lack of detectable anti-KEL antibodies in MZ B cell depleted recipients, anti-IgM and anti-IgG antibodies were not detectable on KEL RBCs transfused into MZ B cell depleted B6 recipients (Figure 4B). Moreover, the decreased level of detectable anti-KEL alloantibodies in MZ B cell depleted B6 recipients correlated with a lack of detectable C3 on transfused KEL-DiI RBCs (Figure 4C), as well as no detectable KEL RBC clearance above that of KEL RBCs transfused into KEL positive recipients (Figure 4D). Taken together, these results suggest that MZ B cells play a key role in regulating a humoral immune response to KEL RBCs following transfusion.

### Follicular B cells are not required for the generation of anti-KEL alloantibodies

**Figure 5:**
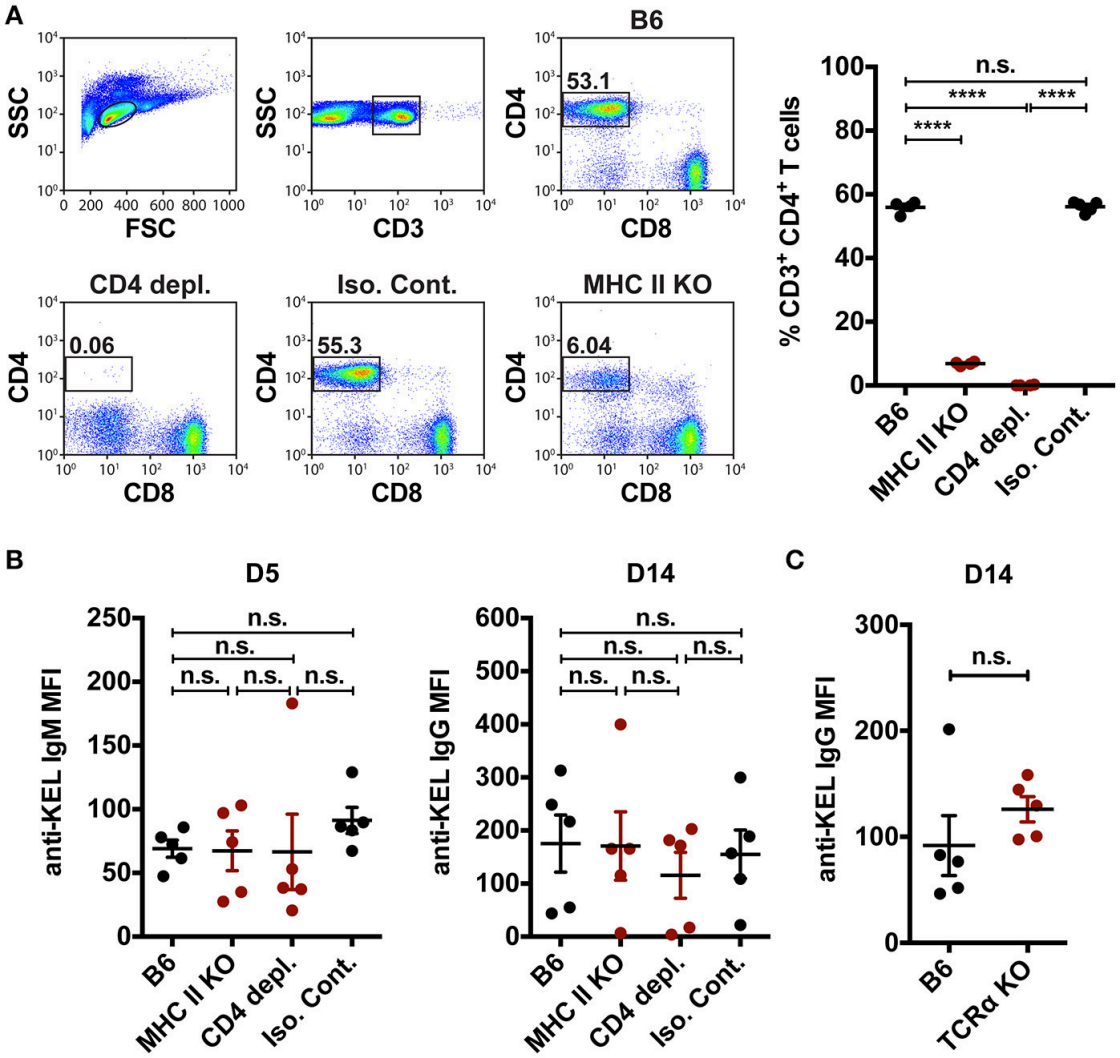
CD4 T cell help is not required to mediate KEL alloimmunization. **(A)** Flow plots and quantification of percent CD3^+^ CD4^+^ CD8^−^ T cells in the spleen of wild type B6 recipients treated with PBS (B6), monoclonal anti-mouse CD4 depleting antibody (CD4 depl.), or Rat IgG2b isotype control antibody (Iso. Cont.), and CD4 T cell deficient MHC Class II KO (MHC II KO) recipients. **(B)** KEL specific alloantibodies in PBS (B6), monoclonal anti-mouse CD4 depleting antibody (CD4 depl.) or Rat IgG2b isotype control antibody (Iso. Cont.) treated B6 recipients and MHC Class II KO (MHC II KO) recipients transfused with KEL RBCs. **(C)** B6 wild type and CD4 T cell deficient TCRα KO recipients were transfused with KEL RBCs and evaluated for anti-KEL IgG formation 14 days (D14) post transfusion. Error bars represent mean ± SEM. The mean of each group is depicted as a horizontal line. Statistics were generated using a one-way ANOVA with Tukey's post-test in panels **(A,B)** or a student *t*-test in panel **(C)**. All panels show representative data from experiments reproduced 3 times, with 5 mice per group per experiment. ^****^p < 0.0001 and n.s. indicates not statistically significant.

In addition to orchestrating rapid production of low affinity IgM following engagement of blood borne antigens, MZ B cells can also coordinate a downstream follicular B cell response that is thought to be ultimately responsible for an optimal immune response to RBC alloantigens. MZ B cells can contribute to canonical follicular B cell responses through potent activation of CD4 T cells (35) and/or trafficking of antigens from the marginal sinus to the follicle, where the antigen can be captured by follicular dendritic cells for presentation to follicular B cells (33). To determine the role of MZ B cells in the formation of alloantibodies to the KEL antigen, we first defined the role of CD4 T cells in the formation of anti-KEL alloantibodies. To accomplish this, MHC Class II knockout (MHC II KO) recipients that are genetically deleted of MHC Class II molecules were transfused with KEL RBCs (Figure 5A). Transfusion of KEL RBCs into MHC II KO recipients resulted in an equivalent level of anti-KEL antibody formation as that of wild type B6 recipients (Figure 5B). As MHC II KO recipients possess significantly reduced but present CD4 T cells (Figure 5A), it remained possible that the residual CD4 T cells present may drive an alloantibody response to KEL independent of MHC class II. To test this, wild type B6 recipients were administered a CD4 depleting antibody (clone: GK1.5) two times a day apart to deplete CD4 T cells (Figure 5A). To assure optimal detection of CD4 T cell depletion, CD4 depletion was examined using an anti-mouse CD4 antibody (clone: RM4-5) that recognizes a completely distinct epitope than the injected CD4 depleting antibody clone GK1.5 (Supplementary Figure 4). Transfusion of KEL RBCs into CD4 T cell depleted recipients also resulted in an equivalent level of anti-KEL antibody formation as that seen in wild type B6 recipients (Figure 5B). As an additional measure to examine the potential requirement of CD4 T cells in the development of anti-KEL IgG following KEL RBC transfusion, we next transfused TCRα KO mice that are genetically deficient in CD4 T cells with KEL RBCs. Similar to MHC class II KO and CD4 T cell depleted recipients, TCRα KO mice generated a similar anti-KEL IgG response as B6 wild type control mice following KEL RBC exposure (Figure 5C). Together, these findings demonstrate that alloimmunization to the KEL antigen following transfusion of KEL RBCs can occur independent of CD4 T cell help, and therefore that MZ B cells do not likely facilitate alloantibody formation through direct or indirect activation of CD4 T cells.

**Figure 6:**
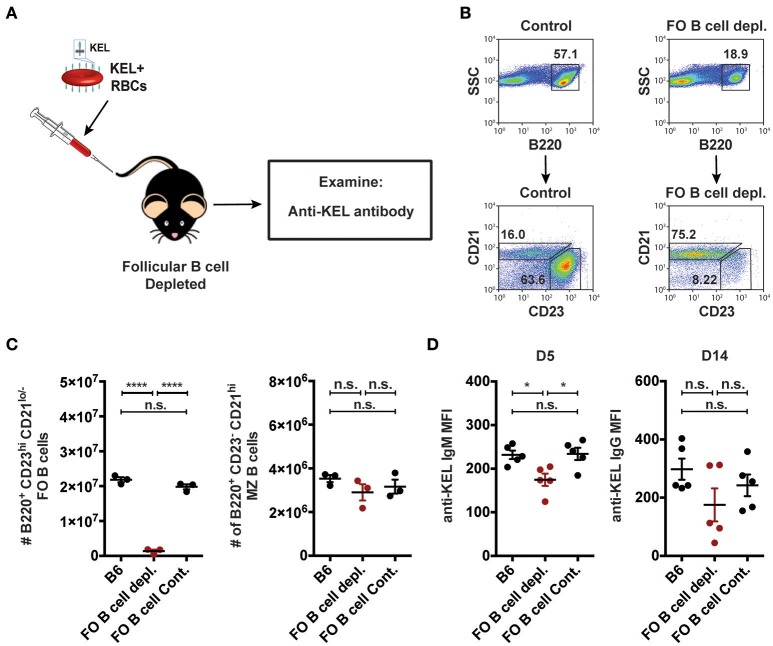
Alloantibodies specific to KEL develop independent of follicular B cells. **(A)** Experimental schematic. B6 recipients negative for KEL were treated with a single tail vein injection of PBS (B6), monoclonal anti-mouse CD20 IgG1 antibody (FO B cell depl.), or mouse IgG1 isotype control antibody (FO B cell Cont.). Recipients were then transfused with KEL RBCs and evaluated for anti-KEL antibodies. **(B)** Gating strategy and dot plots of splenic B220^+^ CD23^+^ CD21^lo/−^ follicular (FO) B cells and B220^+^ CD23^−^ CD21^hi^ MZ B cells in control recipients or monoclonal anti-mouse CD20 IgG1 antibody (FO B cell depl.) treated recipients. **(C)** Quantitation of B220^+^ CD23^+^ CD21^lo/−^ follicular (FO) B cells and B220^+^ CD23^−^ CD21^hi^ MZ B cells in B6 recipients treated with PBS (B6), monoclonal anti-mouse CD20 IgG1 antibody (FO B cell depl.), or mouse IgG1 isotype control antibody (FO B cell Cont.). **(D)** Anti-KEL antibody formation in B6 recipients treated with PBS (B6), monoclonal anti-mouse CD20 IgG1 antibody (FO B cell depl.), or mouse IgG1 isotype control antibody (FO B cell Cont.) 5 (D5) and 14 (D14) days post transfusion of KEL RBCs. Error bars represent mean ± SEM. The mean of each group is depicted as a horizontal line. Statistics were generated using one-way ANOVA with Tukey's post-test. All panels show representative data from experiments reproduced 2 times, with 3 **(C)** or 5 **(D)** mice per group per experiment. ^****^p < 0.0001; ^*^p < 0.05 and n.s. indicates not statistically significant.

As MZ B cells were found to be required for formation of anti-KEL alloantibodies but CD4 T cells were not (Figures 3, 5), we next examined whether MZ B cells can also generate antibodies to the KEL antigen independent of follicular B cells. To do this, we depleted follicular B cells using an anti-CD20 IgG1 depleting monoclonal antibody, which specifically removes follicular B cells while leaving MZ B cells intact (51). B6 recipients negative for KEL were administered PBS (B6), follicular B cell depleting monoclonal antibody anti-CD20 IgG1 (clone: 18B12), or mouse IgG1 isotype control antibody (clone: MOPC-21) 2 weeks prior to transfusion (Figure 6A). Immediately before transfusion, follicular B cell depletion was assessed in the spleen of animals treated in parallel (Figures 6B,C). Following KEL RBC transfusion, serum was collected and evaluated for anti-KEL IgM and IgG. Depletion of follicular B cells did not significantly impact the immunological outcome of KEL alloimmunization, with anti-KEL IgM and IgG alloantibodies detectable at similar levels to PBS (B6) and isotype control treated B6 recipients (Figure 6D). Combined, these findings suggest that anti-KEL antibody formation may occur through a MZ B cell dependent pathway.

### MZ B cells in part utilize IFNAR signaling to induce anti-KEL IgM

**Figure 7:**
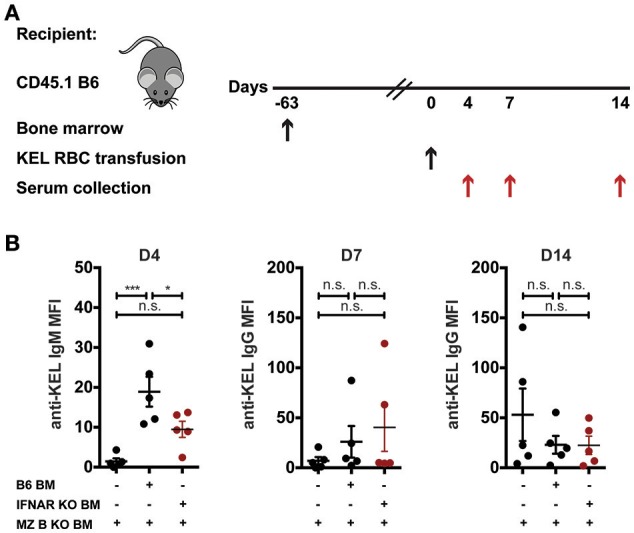
IFNAR on MZ B cells influences anti-KEL IgM antibody formation. **(A)** Experimental schematic. CD45.1 B6 recipients negative for KEL were lethally irradiated and reconstituted with CD45.2 IFNAR KO bone marrow mixed with MZ B cell KO bone marrow to generate MZ B cell specific IFNAR KO recipients. Chimeric recipients were then transfused with KEL RBCs and evaluated for anti-KEL antibodies. **(B)** Anti-KEL antibody formation in chimeric recipients 4 (D4), 7 (D7), and 14 (D14) days post transfusion of KEL RBCs. Error bars represent mean ± SEM. The mean of each group is depicted as a horizontal line. Statistics were generated using one-way ANOVA with Tukey's post-test. All panels show representative data from experiments reproduced 2 times, with 5 mice per group per experiment. ^***^p < 0.001; ^*^p < 0.05 and n.s, not statistically significant.

Recent data suggest that B cells require type I interferons to produce alloantibodies to the KEL antigen on transfused RBCs (59). As a result, we next examined whether MZ B cells specifically require type I interferon signaling to induce an alloantibody response to KEL RBCs. To accomplish this, we generated chimeric recipients that specifically lack type I interferon alpha receptor (IFNAR) expression on MZ B cells. To do this, CD45.1 B6 recipients were lethally irradiated and reconstituted with a mixture of MZ B cell KO bone marrow and B6 wild type or IFNAR KO bone marrow. Recipients reconstituted with only MZ B cell KO bone marrow were included as a control for the potential impact of MZ B cell KO bone marrow reconstitution. Following reconstitution, recipients were transfused with KEL RBCs and examined for KEL alloimmunization (Figure 7A). While recipients producing IFNAR expressing MZ B cells generated a significant anti-KEL IgM response to KEL RBCs compared to recipients that are deficient in MZ B cells, the lack of IFNAR expression on MZ B cells resulted in a dampened IgM response to KEL on transfused RBCs (Figure 7B). Conversely, the anti-KEL IgG response in recipients deficient in IFNAR expression on MZ B cells was statistically similar to the IgG response observed in B6 recipients reconstituted with only MZ B cell KO bone marrow and MZ B cells that express IFNAR (Figure 7B).

## Discussion

RBC alloimmunization can result in significant increases in morbidity and mortality in transfusion-dependent patients (2–5, 7, 8). Although antigen-matching protocols reduce RBC alloimmunization, recent studies demonstrate that these approaches often fail to adequately prevent the formation of clinically relevant alloantibody formation (11). Unfortunately, there are currently no available strategies to actively prevent alloantibody formation following therapeutic RBC transfusion. This in part reflects a lack of understanding regarding key factors responsible for initiating RBC alloimmunization. Thus, identification of key immune constituents involved in the generation of antibody responses to donor RBC antigens is pivotal to the development of novel strategies to prevent RBC alloimmunization.

In order to dissect key factors responsible for regulating RBC alloantibody formation, we employed a murine model of RBC alloimmunization. Using this approach, our results demonstrate that KEL RBCs appear to co-localize with MZ B cells and that depletion of MZ B cells prevents alloantibody formation following KEL RBC transfusion. Taken together, these results suggest that MZ B cells may play a key role in the development of alloantibodies following RBC transfusion, and therefore may represent a distinct target in the prevention of this process. While MZ B cells have been shown to traffic antigens from the marginal sinus to the follicle, where follicular dendritic cells can capture the antigen for presentation to follicular B cells and subsequent generation of germinal center reactions (33, 73), MZ B cells themselves have also been shown to directly contribute to class switched antibody production (32, 74). Consistent with this, MZ B cell depletion completely prevented RBC alloantibody formation, while analogous removal of follicular B cells failed to similarly impact the humoral immune response to KEL. Given the ability of KEL RBCs to drive anti-KEL antibody formation in the absence of CD4 T cells or follicular B cells, these results suggest that MZ B cells may directly contribute to KEL alloimmunization through generation of anti-KEL IgG following KEL RBC exposure. These findings do not exclude the possibility that when present follicular B cells may also participate in the humoral immune response to KEL; the alloantibody response to KEL RBCs was lower in the absence of follicular B cells, though not statistically different. Rather, the current study demonstrates that both follicular B cells and MZ B cells likely play a role in the total anti-KEL IgG response to KEL RBCs.

MZ B cells exhibit a lower activation threshold, an attribute that presumably reflects high basal expression of germ-line encoded receptors, including toll-like receptors and complement receptors (CD21/CD35). The lower threshold required for MZ B cell activation may in part explain why RBC alloantigens possess the capacity to drive alloantibody formation in the absence of known adjuvants. Given the role of MZ B cells in alloantibody formation to KEL, similar activation requirements may not be necessary to induce antibody formation in the absence of known secondary activation signals, as recently demonstrated for other antigens (49). Furthermore, given a recent study demonstrating that the density of a RBC alloantigen can impact the development of alloantibodies to RBC antigens (56), optimal presentation of antigen density on the RBC surface may in part account for the ability of KEL RBCs to drive alloantibody formation through a MZ B cell dependent but CD4 T cell independent process. Moreover, in addition to antigenic density, a recent study demonstrates that B cells necessitate type I interferons to generate alloantibodies to KEL (59), suggesting that type I interferon expression on MZ B cells may contribute to the ability of MZ B cells to directly produce anti-KEL IgG in the absence of CD4 T cell help. Recipients deficient in type I interferon (IFNAR) expression specifically on MZ B cells generated a diminished anti-KEL IgM response to KEL RBCs. While these results suggest that IFNAR signaling on MZ B cells may play a role in the initial generation of anti-KEL antibodies following KEL RBC exposure, the formation of anti-KEL IgG appeared to be unaffected. However, as the MZ B KO donors utilized to generate these chimeric recipients were not 100% deficient in MZ B cells, it is possible that the recipients reconstituted with IFNAR KO and MZ B cell KO bone marrow may actually possess a low frequency of IFNAR expressing MZ B cells. Furthermore, the overall blunted anti-KEL IgG response in bone marrow transplanted recipients in general makes it more difficult to interpret these data. Thus, these data reflect the inherent limitations of currently available tools and do not rule out the possibility that type I interferons may play a role in MZ B cell-mediated antibody production. As no model lacks complete MZ B cell genetic ablation in the absence of other immune defects, discerning the exact role of type I interferons in activation of MZ B cells for IgG production remains difficult. Moreover, as no B cell receptor transgenic or B cell tetramer for KEL currently exist, it is difficult to elucidate the exact mechanism by which KEL directly activates MZ B cells for IgG production. Nonetheless, the ability of MZ B cells to induce alloantibody formation does not rule out the possibility that transfused KEL RBCs can activate a follicular B cell response and that these cells may likewise contribute to KEL alloimmunization under certain conditions; consistent with this, while not statistically significant, depletion of follicular B cells resulted in a trend toward a reduced alloantibody response following KEL RBC transfusion. However, these data do suggest that MZ B cells are required for RBC alloimmunization to KEL and that in the absence of follicular B cells, MZ B cells appear to be sufficient to provide an immune response to KEL RBCs.

While some differences certainly exist between human and mouse splenic architecture (14), numerous studies have demonstrated that MZ B cells play the same fundamental role in mouse and human spleens: these cells trap and immediately respond to encountered antigen, followed by trafficking antigen to key immune compartments (28, 29, 33, 75). These results suggest that MZ B cells may likewise mediate RBC alloimmunization in patients, and may therefore represent a unique target that could be used to prevent RBC alloantibody formation in chronically transfused patients. Consistent with this, the only prospective study examining immune responses to RBC antigens in splenectomized humans demonstrated that the spleen was absolutely required for anti-RBC antibody formation (67), suggesting that splenic constituents are key players in the development of an alloantibody response to RBC antigens following transfusion. A recent large retrospective study examining RBC alloimmunization in splenectomized patients corroborated these earlier findings (68), once again suggesting that the spleen plays a central role in mediating RBC alloantibody formation. While the splenic microenvironment is difficult to recapitulate *in vitro*, given the distinct localization and activity of MZ B cells within the spleen and the location of the spleen with the circulatory system, these results strongly suggest that similar engagement of allogeneic antigens on transfused RBCs by MZ B cells within the spleen of transfused patients likely represents a key early step in this process.

While patients with transfusion-dependent conditions, such as sickle cell disease, may often have altered spleen function and may even be considered “functionally asplenic,” recent studies suggest that most sickle cell patients actually possess residual spleen function, which can be significantly increased following hydroxyurea therapy or RBC transfusion, common treatment modalities for this patient population (76–81). As murine and human MZ B cells differ in that human MZ B cells circulate and are present in the spleen as well as other secondary lymphoid organs, while murine MZ B cells demonstrate a low-circulative capacity and are restricted to the spleen, it is also possible that under certain conditions, such as altered spleen function, extrasplenic MZ B cells may be involved in responding to RBC alloantigens. Thus, while the ability to directly extrapolate our findings to a clinical setting remains to be determined, the findings of the present study provide potential insight into a key immune pathway of alloimmunization to RBC antigens.

While MZ B cells may potentially serve as a future pharmacological target in the prevention of RBC alloimmunization, the requirement of complete MZ B cell depletion for effective prevention of alloimmunization suggests that a combinatorial approach targeting various aspects of MZ B cell biology may be required, if this approach is to ever be realized clinically. However, with a potential immunological target identified, complementary approaches to favorably manipulate MZ B cell function can now be the focus of future studies seeking to prevent RBC alloimmunization in transfusion-dependent patients. In addition to providing a potential pharmacological target, the involvement of MZ B cells in RBC alloimmunization may also offer important insight into another fundamental question with transfusion immunology. Although a significant number of chronically transfused patients develop alloantibodies, not all patients become alloimmunized to transfused donor RBC alloantigens. Individuals that generate alloantibodies to transfused RBC antigens following RBC transfusion are referred to as “responders,” while patients who fail to become alloimmunized following RBC alloantigen exposure are often called “non-responders” (3, 82). Several studies indicate that the precursor frequency of antigen specific T cells can predict the immunological response even within inbred strains of mice (83–86), suggesting that variations in the frequency of antigen specific cells may represent a key regulator of an immune response to a given antigen. As MZ B cells possess a restricted and distinct repertoire of antibody specificities (32, 87–90), these data suggest that differences in the precursor frequency of MZ B cells against a given alloantigen within a patient may predict the likelihood that an individual may develop alloantibodies following transfusion. Therefore, in addition to the potential impact of genetic or other environmental modifiers of general immune function (91–102), differences in the frequency of RBC alloantigen specific MZ B cells may represent an additional contributing factor that influences the likelihood of alloantibody formation following RBC transfusion.

In summary, the present data demonstrate that MZ B cells represent a previously underappreciated immune constituent involved in RBC alloimmunization. The relevancy of the current findings to human medicine is that they not only suggest a unique immunological pathway by which RBC alloimmunization occurs but also a potential immunological target that may aid in preventing and possibly predicting RBC alloimmunization.

## Author contributions

SP, DG, JH, and SS designed the research study. SP carried out and analyzed experiments together with DG, KG-P, XZ, LR, RJ, MF, AB, and NS. CM, CA, PZ, SC, CT, and JH provided critical support. SP and SS wrote the manuscript, which was additionally edited and commented on by the others.

### Conflict of interest statement

The authors declare that the research was conducted in the absence of any commercial or financial relationships that could be construed as a potential conflict of interest.

## Abbreviations

MZ B cells: marginal zone B cells
KEL: Kell RBCs.
