# Supplementary material for: Marginal Zone B Cells Induce Alloantibody Formation Following RBC Transfusion
Source: Front Immunol. 2018 Nov 16;9:2516. doi: 10.3389/fimmu.2018.02516 (PMC6250814; doi:10.3389/fimmu.2018.02516)
Supplement: Supplementary file 1 [file Data_Sheet_1.docx]

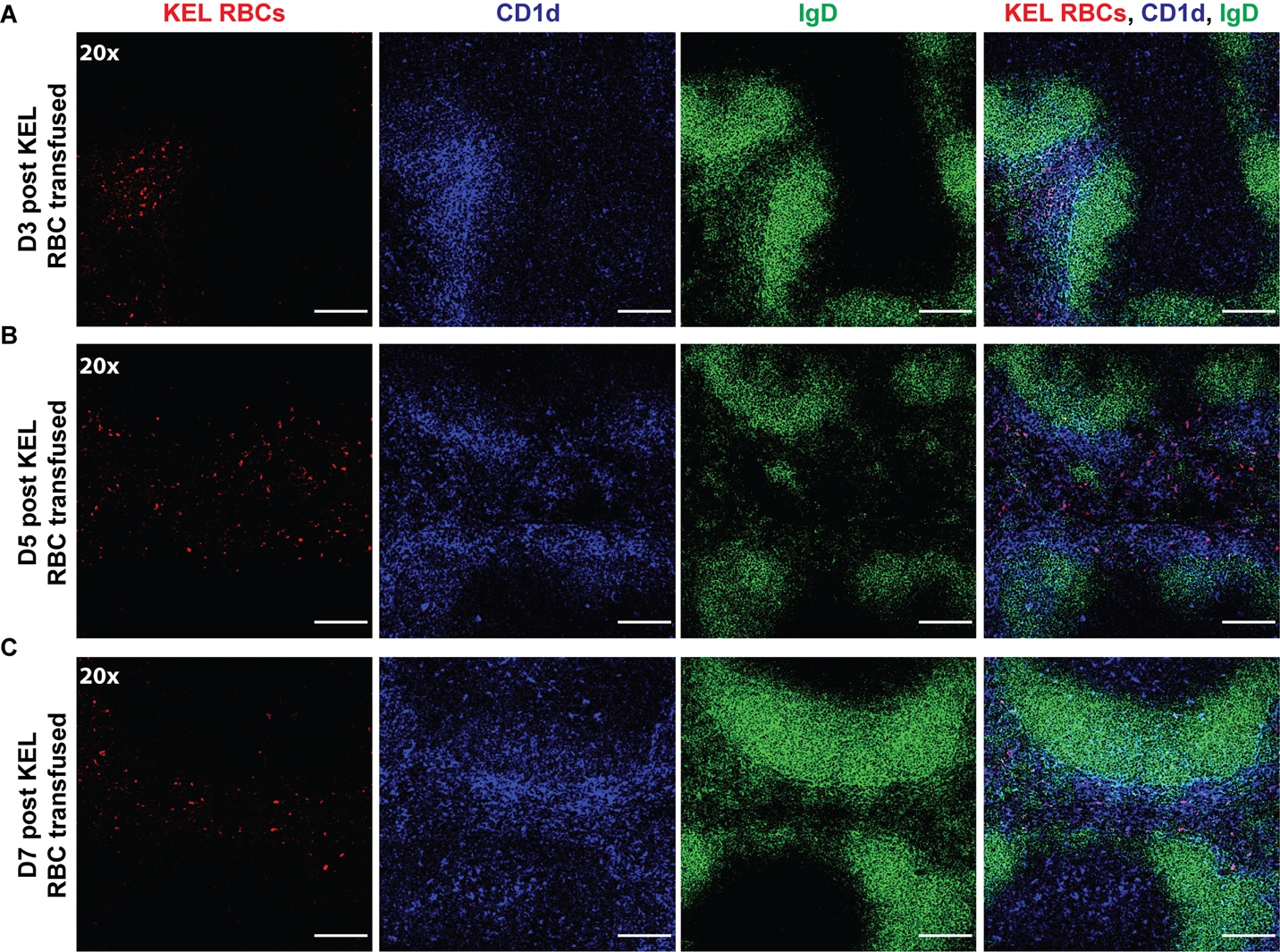


**Supplementary Figure 1. KEL-DiO RBCs are not detectable in the follicle 3, 5 and 7-days post transfusion.** B6 recipients negative for KEL were transfused with KEL-DiO RBCs **(**red**)**, followed by confocal analysis of KEL RBC localization **(A)** 3, **(B)** 5 and **(C)** 7 days post transfusion. MZ B cells are identified as IgD (green) dim and CD1d (blue) bright, while follicular B cells are distinguished as IgD (green) bright and CD1d (blue) dim. Samples were analyzed using a 20x objective. Scale bar = 100 μm. All panels show representative data from experiments reproduced 2 times, with 3 mice per group per experiment.
