# Supplementary material for: Marginal Zone B Cells Induce Alloantibody Formation Following RBC Transfusion
Source: Front Immunol. 2018 Nov 16;9:2516. doi: 10.3389/fimmu.2018.02516 (PMC6250814; doi:10.3389/fimmu.2018.02516)
Supplement: Supplementary file 2 [file Data_Sheet_2.docx]

**Supplementary Figure 2. MZ B cell depletion does not negatively impact the percent frequency of follicular B cells and T cells.** B6 recipients were administered 2 intra-peritoneal injections of PBS (B6), a MZ B cell depleting antibody cocktail (mouse monoclonal anti-mouse CD11a + mouse monoclonal anti-mouse CD49d) or an isotype control antibody cocktail (Rat IgG2b + Rat IgG2a). One day following the last injection, splenocytes were harvested and examined for **(A)** follicular B cells (B220^+^ CD23^+^ CD21^lo/-^), **(B)** CD4 (CD4^+^ CD8^-^) or CD8 (CD4^-^ CD8^+^) T cells, **(C)** macrophages (CD11b^+^ F4/80^+^), **(D)** dendritic cells (DCs; CD11c^+^ I-A/E^b+^) or **(E)** neutrophils (CD11b^+^ Gr1^+^). Errors bars represent mean + SEM. Statistics were generated using a one-way ANOVA with a post Tukey’s multiple comparison test. *** p< 0.001 and n.s. indicates not statistically significant.
