# Supplementary material for: Marginal Zone B Cells Induce Alloantibody Formation Following RBC Transfusion
Source: Front Immunol. 2018 Nov 16;9:2516. doi: 10.3389/fimmu.2018.02516 (PMC6250814; doi:10.3389/fimmu.2018.02516)
Supplement: Supplementary file 3 [file Data_Sheet_3.docx]

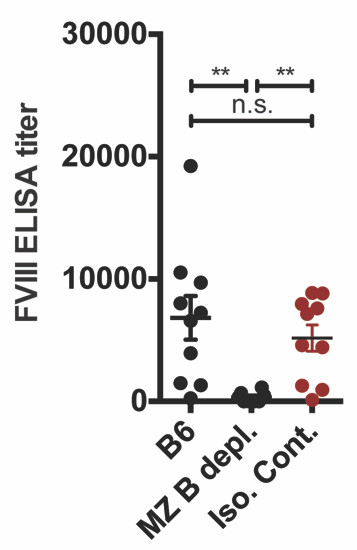


**Supplementary Figure 3. MZ B cell depletion prevents formation of anti-FVIII antibodies.** Antibody formation against FVIII following FVIII exposure in B6 recipients administered PBS (B6), a MZ B cell depleting antibody cocktail (mouse monoclonal anti-mouse CD11a + mouse monoclonal anti-mouse CD49d) or an isotype control antibody cocktail (Rat IgG2b + Rat IgG2a). Errors bars represent mean + SEM. Statistics were generated using a One-way ANOVA with a post Tukey’s multiple comparison test. ** p< 0.01 and n.s. indicates not statistically significant.
