# Supplementary material for: Marginal Zone B Cells Induce Alloantibody Formation Following RBC Transfusion
Source: Front Immunol. 2018 Nov 16;9:2516. doi: 10.3389/fimmu.2018.02516 (PMC6250814; doi:10.3389/fimmu.2018.02516)
Supplement: Supplementary file 4 [file Data_Sheet_4.docx]

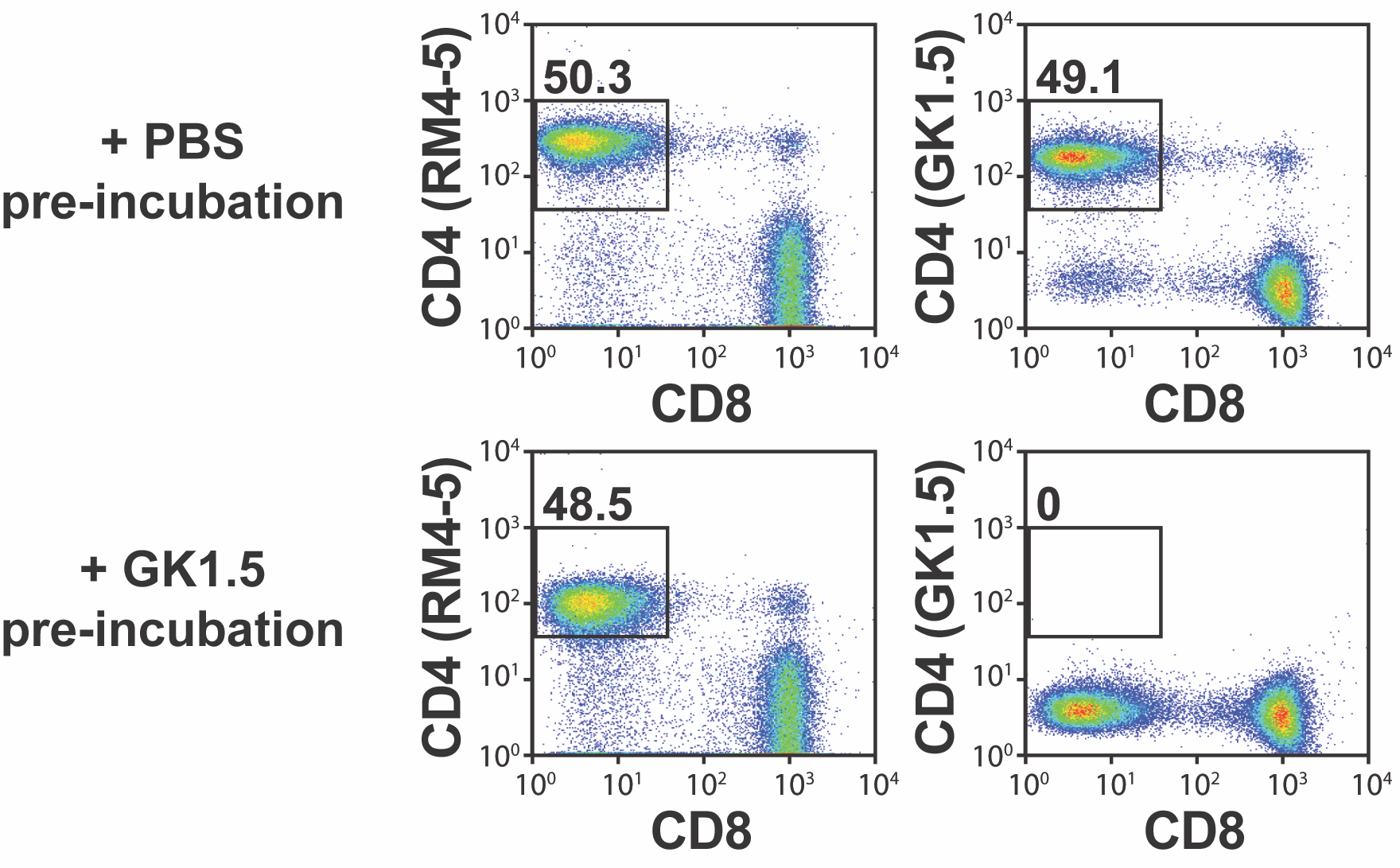


**Supplementary Figure 4. Detection of CD4^+^ T cells following administration of an anti-CD4 depleting antibody (clone: GK1.5).** Representative flow plots of percent CD3^+^ CD4^+^ CD8^-^ T cells following *in vitro* treatment of PBS or monoclonal anti-mouse CD4 depleting antibody (GK1.5). Following incubation with PBS or the GK1.5 anti-mouse CD4 depleting antibody, samples were stained with APC rat anti-mouse CD4 (clone RM4-5) + BV605 rat anti-mouse CD4 (clone GK1.5) + FITC rat anti-mouse CD3 + PE rat anti-mouse CD8 + V500 rat anti-mouse B220.
